# Controlled graphene oxide assembly on silver nanocube monolayers for SERS detection: dependence on nanocube packing procedure

**DOI:** 10.3762/bjnano.7.2

**Published:** 2016-01-06

**Authors:** Martina Banchelli, Bruno Tiribilli, Roberto Pini, Luigi Dei, Paolo Matteini, Gabriella Caminati

**Affiliations:** 1 Institute of Applied Physics, National Research Council - Via Madonna del Piano 10, I-50019 Sesto Fiorentino, Italy; 2 Institute for Complex Systems, National Research Council, Via Madonna del Piano 10, I-50019 Sesto Fiorentino, Italy; 3 Department of Chemistry and CSGI, University of Florence, Via della Lastruccia 3–13, I-50019 Sesto Fiorentino, Italy

**Keywords:** graphene oxide, quartz crystal microbalance, sensing application, SERS, silver nanocubes

## Abstract

Hybrid graphene oxide/silver nanocubes (GO/AgNCs) arrays for surface-enhanced Raman spectroscopy (SERS) applications were prepared by means of two procedures differing for the method used in the assembly of the silver nanocubes onto the surface: Langmuir–Blodgett (LB) transfer and direct sequential physisorption of silver nanocubes (AgNCs). Adsorption of graphene oxide (GO) flakes on the AgNC assemblies obtained with both procedures was monitored by quartz crystal microbalance (QCM) technique as a function of GO bulk concentration. The experiment provided values of the adsorbed GO mass on the AgNC array and the GO saturation limit as well as the thickness and the viscoelastic properties of the GO film. Atomic force microscopy (AFM) measurements of the resulting samples revealed that a similar surface coverage was achieved with both procedures but with a different distribution of silver nanoparticles. In the GO covered LB film, the AgNC distribution is characterized by densely packed regions alternating with empty surface areas. On the other hand, AgNCs are more homogeneously dispersed over the entire sensor surface when the nanocubes spontaneously adsorb from solution. In this case, the assembly results in less-packed silver nanostructures with higher inter-cube distance. For the two assembled substrates, AFM of silver nanocubes layers fully covered with GO revealed the presence of a homogeneous, flexible and smooth GO sheet folding over the silver nanocubes and extending onto the bare surface. Preliminary SERS experiments on adenine showed a higher SERS enhancement factor for GO on Langmuir–Blodgett films of AgNCs with respect to bare AgNC systems. Conversely, poor SERS enhancement for adenine resulted for GO-covered AgNCs obtained by spontaneous adsorption. This indicated that the assembly and packing of AgNCs obtained in this way, although more homogeneous over the substrate surface, is not as effective for SERS analysis.

## Introduction

Organized films composed of metal nanoparticles have been extensively studied in recent years owing to their enormous potential in fields as diverse as photoelectrochemistry [1–2], optoelectronics [3], energy-harvesting applications [4], cancer imaging and therapy [5], sensing and biosensing applications [6–7]. In particular, sensors based on arrays of noble metal nanoparticles have become increasingly popular for the ultrasensitive detection of a variety of species ranging from small molecules to large proteins by means of surface-enhanced Raman spectroscopy (SERS) [8–9]. Furthermore, these arrays offer additional sensing capabilities based on the localized surface plasmon resonance (LSPR) sensitivity to subtle changes in the refractive index of the surrounding molecular environment [10–11].

Nanoparticle arrays differing in chemical composition, size, shape and bidimensional morphology have been extensively studied [8–9,12] in the past decades. The existing literature has revealed that not only the shape and size of metal nanoparticles determine their physicochemical and optical properties but also their bidimensional packing affects their properties. Among others, silver nanocubes (AgNCs) have been demonstrated to provide an intense and reproducible amplification of the Raman signal when densely assembled in ordered 2D structures on solid supports [13–15]. The large SERS effect has been demonstrated to be strictly dependent on the gap distance of adjacent nanostructures, commonly termed "hot spots", and many different approaches have been proposed for their production. Early methods rely on the random aggregation of silver or gold nanoparticles induced by a salt [16] whereas more recently external magnetic field were employed to dynamically control the interparticle spacing of a nanoparticle monolayer at the hexane/water interface [17]; however, the fabrication of controllable hot spots still remains a remarkable challenge.

The outstanding SERS capability of metal nanoparticle arrays may be further extended by a proper pairing with graphene or graphene derivatives due to their exclusive chemical, electronic and mechanical properties [18]. Graphene oxide (GO) is derived in the form of single-atom sheets of conjugated sp^2^ carbon atoms with abundant oxygen-containing functionalities, which confer to the system additional features including great chemical stability in aqueous media and superior ability of capturing and retaining molecules.

Reports on nanoparticle/graphene hybrid nanocomposites showed that SERS signals arising from graphene/metal hybrid structures are higher compared to those of the individual components [19]. Several methods have been proposed for the fabrication of hybrid composites incorporating plasmonic nanoparticles and graphene [20]; preliminary results from this group evidenced that large SERS enhancement factors were obtained for rhodamine 6G adsorbed on a combination of graphene oxide and AgNCs arrays [21]. In a closely related paper by our group [22], we investigated the influence of thickness and structuring of the graphene oxide layer covering a Langmuir–Blodgett film of silver nanocubes on SERS detection, in the same paper [22] we compared the experimental results with theoretical simulations obtained by a finite element method (FEM).

In the present paper, we adopt a previously reported procedure for AgNC preparation [22] but we systematically explore the interplay between graphene oxide coverage and the morphology of the underlying AgNC arrays and how the resulting differences in the structural features of the hybrid system affect the spectroscopic properties and eventually SERS enhancement. Different approaches have been explored to assemble nanoparticles, including vacuum deposition, electrochemical deposition, electrostatic layer-by-layer adsorption and formation of nanoparticle films at the liquid−liquid interface [23–26]. In the latter case, assembly of uncapped nanoparticles generally leads to the formation of loosely packed aggregates and linking functionalities must be employed to decrease the interparticle distance, which in turns results in rigid arrays with suppressed elasticity and scarce resistance to mechanical stress. Nevertheless, the fabrication of large-scale homogeneous layers required for SERS detection has not been fully achieved. In this work we assembled closely packed AgNCs arrays with two different approaches: Langmuir–Blodgett (LB) transfer onto the solid support of a floating monolayer of AgNCs (procedure A) and sequential self-assembly of AgNCs by physisorption onto the surface (procedure B). The standard LB technique implies the preparation of a stable floating monolayer at liquid–air interface followed by controlled transfer onto the surface of a solid substrate [24]. The LB procedure was already employed in a related paper [22] for the deposition of AgNCs onto solid substrates, here we extended the preparation protocol investigating in detail the influence of hysteresis effect and monolayer fluidity on the packing of the resulting LB film. The monolayer is prepared by deposition and compaction of the nanoparticles onto water surface, to this aim different approaches can be chosen depending on particle functionalization: particles surrounded by a hydrophobic ligand shell can be deposited directly onto water surfaces [27] either alone or mixed with organic molecules that act as dispersants in the case of scarce particle stability at the interface [28]. Other strategies include choice of non-aqueous monolayer subphases [29] and formation of the monolayer at water–oil interfaces [27]. Although the encouraging successful results in SERS amplification of LB samples, reports on the fabrication procedure are often contradictory and fine experimental control of the resulting structure is not always satisfactory. Self-assembly of metal nanoparticles through surface adsorption has received much less attention [30–32] due to the lack of direct monitoring of the assembly process in situ. In this work, we followed the formation of an adlayer of AgNCs on silicon oxide surfaces by means of a quartz crystal microbalance with dissipation monitoring as a function of time and AgNCs concentration, obtaining information on the kinetics and the mechanism of adsorption as well as the thickness and viscoelastic properties of the 2D structure at surface saturation.

Coverage of the resulting nanocube arrays with a graphene oxide layer was accomplished by spontaneous adsorption of GO. Although fabrication of GO-covered nanoparticles has been explored using several methods such as voltammetric co-reduction [33], formation of composite graphene oxide/PAMAM–silver nanoparticles through self-assembly followed by microwave irradiation [34] and GO drop-casting onto amino-functionalized Ag nanoparticles [19], detailed information on the GO coating step is not systematically studied and validated. Since the Raman scattering enhancement is strictly dependent on the geometry of the system at the nanoscale, controlling how GO affects the distribution of the AgNCs arrays is a key-step for the realization of efficient SERS substrates. Here we monitored the adsorption of GO onto AgNCs with a controlled step-by-step strategy by direct QCM monitoring of the adsorption process, a method that revealed both the mechanism and kinetics of the composite formation onto the AgNCs-covered SiO_2_ surface. The hybrid samples were further characterized by means of atomic force microscopy that differences in the local morphology of the GO/AgNCs clusters.

The relationship between surface coverage and morphology and SERS activity of the GO/AgNCs hybrid structures was addressed characterizing the SERS behaviour of an extensively studied model probe such as adenine adsorbed on the resulting arrays. Preliminary results show that higher SERS intensities are detected from the GO/AgNCs hybrid nanostructures as compared to pure Ag nanoparticles for both nanoparticle packing procedures. Interestingly, we found that although similar GO coverage was found for both systems, which leads to similar quantities of adsorbed probe, the morphology of the nanoparticle layer dictates the effective Raman enhancement behaviour.

## Results and Discussion

### Assembly of silver nanocube monolayers

Silver nanocubes (AgNCs) were synthesized through a polyol synthesis, in the presence of poly(vinylpyrrolidone) (PVP) as a stabilizing agent, adopting an established literature protocol [35]. The resulting nanocube samples were mainly monodisperse with 45 nm average size and contained only a negligible fraction of Ag rods and irregular aggregates [36]. AgNC arrays were prepared with two different approaches: Langmuir–Blodgett (LB) transfer onto the solid support of a floating monolayer of AgNC (procedure A) and sequential self-assembly of AgNCs by physisorption onto the surface (procedure B).

#### Procedure A. Controlled assembly of AgNCs by Langmuir–Blodgett technique

**Figure 1:**
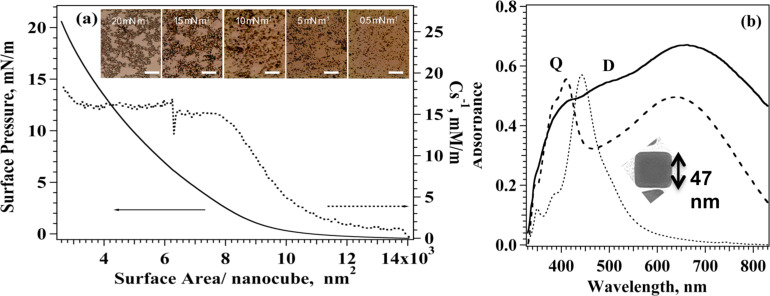
Procedure A. Formation of AgNC arrays by means of the Langmuir–Blodgett technique. (a) π–A and C_s_^−1^–A isotherm for AgNCs at the water–air interface. Optical images (650 × 750 µm) of the LB film of AgNC transferred at different surface pressures along the isotherm. (b) Absorption spectra of AgNC dispersion (dotted line) of a 1 LB layer of AgNC transferred onto glass at 15 mN/m (dashed line) and 20 mN/m (solid line). Inset: Transmission electron microscopy image of a representative single AgNC.

The dispersion of silver nanocubes was spread at the water–air interface from a chloroform solution in a Langmuir trough to fabricate a monolayer of Ag nanoparticles. In agreement with the pioneering work by El-Sayed [37], we found that AgNCs are easily spread at the water–air interface to obtain ordered floating monolayers, in addition we observed that the shape and position of π–A isotherms strongly depend on factors such as the time allowed for solvent evaporation before starting compression, the amount of substance spread at the interface and the compression speed. Careful optimization of these parameters generated reproducible isotherms up to π = 20 mN/m, compression beyond this value leads to unstable monolayers due to incipient collapse of the film and formation of 3D structure. A typical π–A isotherm for AgNCs is reported in Figure 1a together with the behaviour of the surface compressional modulus as a function of surface area.

Surface pressure was found to increase monotonically as available surface area decreases showing a subtle phase transition more evident after a first expansion–compression cycle (see Supporting Information File 1, Figure S1) located at π = 4 mN/m in contrast with what reported previously [29,37]. We computed the surface compressional modulus from the experimental π–A data using Equation 1

[1]
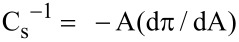


The surface compressional modulus is related to the elasticity and fluidity of the monolayer [38] and its value identifies the different monolayer phases: C_s_^−1^ values lower than 50 mN/m correspond to a liquid-expanded phase, whereas for highly condensed phases values as high as 300 mN/m can be observed. Interestingly, the C_s_^−1^ value remains constant as surface pressure increases revealing the presence of a wide domain where the mechanical features of the film remain unchanged. The small C_s_^−1^ values described in Figure 1a, reveal fluid and elastic phase of the AgNC monolayer, similar low values of the surface compressional modulus (C_s_^−1^ < 50 mN/m) were found for nanoparticles trapped at the air–water interface also by other authors [39] who demonstrated the underlying correlation between the observed macroscopic transitions in mechanical properties and the microscopic dynamical phase transitions. Compression-expansion cycles were performed on AgNC monolayers arresting compression below 20 mN/m, the results (reported in Supporting Information File 1, Figure S1) show a small hysteresis that vanishes completely after the second cycle. These findings exclude loss of material in the subphase upon compression and support the formation of elastic arrays of AgNCs that quickly recover their closely-packed morphology after expansion thanks to the presence of the PVP polymer surrounding the nanoparticles. These features also warrant a successful transfer of the film from the water subphase to the solid support (Supporting Information File 1, Figure S2), fast reorganization of the nanocubes at the interface when the material is transferred to the solid substrate is evidenced by the stable value of surface pressure along the transfer process. Langmuir–Blodgett layers were transferred onto glass and silicon supports at different target surface pressures spanning from 0.5 mN/m to 20 mN/m (see Figure 1a), the transfer ratio (Supporting Information File 1, Figure S2), which reports on the quality of the transferred layer, was homogenous over the entire surface and close to one for all the selected surface pressures. Interestingly, conventional amphiphiles cannot be transferred successfully at low surface pressure whereas for AgNC significant transfer was obtained also at 0.5 mN/m although with low nanocube density. Such behaviour, reported also by other authors for mixed AgNC/phospholipid systems [40], is likely due to the stabilizing effect of PVP cushion capping the surface of the nanocubes.

Optical microscopy in reflection mode of LB monolayers of AgNCs transferred on silicon oxide, reported in Figure 1a, shows that as the transfer surface pressure increases, the particle density and the average size of the nanocube clusters increase, until a near-continuous monolayer is established around 15 mN/m. Images of the sample collected at 20 mN/m reveal the presence of 3D clusters of nanocubes supporting the hypothesis of incipient monolayer collapse at this surface pressure.

The principal structural features characterizing the AgNC assembly obtained with procedure A are summarized in Table 1 together with the results obtained for procedure B.

**Table 1:** Structural parameters for AgNC arrays on silicon oxide.

AgNC packing procedure	Surface density, NC/µm^2^	Interparticle distance, nm	δ, nm	Δ*D* × 10^−6^

procedure A (π_transfer_ = 15 mN/m)	41^a^	100^a^	50^b^	—
procedure B, sequential adsorption	35^a^	119^a^	32 ± 10^a^	18^a^

^a^Values extracted from analysis of QCM measurements. ^b^Values extracted from analysis of AFM data.

The data show that the LB layer transferred at 15 mN/m has an average thickness δ = 50 nm demonstrating the formation of a single layer of silver nanotubes. The surface density determined for QCM measurements for LB transferred directly on the QCN sensor was 41 NC/µm^2^ which resulted in an average interparticle distance over the entire sensor surface of 100 nm, we recall that direct measurement of nanocube density at the water–air interface is vitiated by the presence of capping PVP molecules which cannot be directly quantified. Local interparticle distance estimated by AFM results [40] evidenced much smaller gaps of 1–3 nm between face-to-face nanocubes nm within the AgNC clusters.

Extinction spectra were collected for all transferred samples. Two typical spectra of 1 LB layer transferred at 15 and 20 mN/m together with the spectrum obtained for the dispersion of AgNCs are reported in Figure 1b. AgNC dispersion exhibits a sharp peak around 450 nm that, according to previous reports [41–42], can be ascribed to the LSPR of nanocubes with 50 nm edge size in agreement with our TEM results and our preliminary findings [21] on different nanocube dispersions. Two minor peaks (348 and 380 nm) are also observed for the disperse NCs, likely due to the small fraction of Ag particles of different size and shape in the dispersion as reported earlier [42]. Spectra obtained for LB monolayers transferred at 15 and 20 mN/m exhibited a red-shifted shoulder at 490 nm together with the appearance of a new peak at 412 nm and a broader signal centred at 640 nm; similar spectral features were already reported for metal nanoparticles on dielectric support [31,43].

These experimental and theoretical studies demonstrate that the degeneracy of the localized surface plasmon resonance (LSPR) mode is split in two orthogonal electron oscillations with respect to the surface plane when a strong near-field interaction between AgNPs occurs in the 2D array. The cubic geometry of the nanoparticles provides a large nanoparticle–substrate contact area, leading to efficient hybridization of dipolar (D) and quadrupolar (Q) plasmonic resonances, which appear in the spectra as two separate D and Q peaks with the new Q band blue-shifted with respect to the D band due to dipolar modes. Therefore, a regular Ag nanocube array is expected to show an intense quadrupolar resonance and a dipolar red-shift relative to the solution of AgNCs, and this effect can be enhanced, or quenched, by controlling the particle size, the surrounding dielectric medium and the interparticle distance [27], namely the Q band is enhanced as the interparticle distance in the AgNC array decreases [44]. Other groups studied LB arrays of DOPC/AgNC of similar sizes [43] and AgNP assembly transferred from the hexane/water interface [44], these authors assigned the signal observed at 414 nm and 390 nm to quadrupolar coupling modes and observed a red shift of the dipolar contribution in agreement with our results. We also observed the appearance of a broad intense band due to strong interparticle dipole–dipole coupling centred at 642 nm and 665 nm for LB films transferred at 15 mN/m and 20 mN/m, respectively. Experimental and theoretical studies [45] on two dimensional (2D) arrays of AgNPs with the different edge-to-edge distances showed that delocalized long range LSPR results in a broad band centred at 640 nm for interparticle distance *d* = 3 nm and that the band red-shifts with increasing *d.* Although a definite assignment in the short wavelength region is hindered by the superposition of the signal of non-cubic aggregates to quadrupolar bands, our results show that regular arrangement of close-packed AgNPs contributes to the efficient coupling of dipole modes and that such coupling is less efficient for samples transferred at 20 mN/m, likely due to crystalline fusion in the collapsed 3D microdomains found in this samples. These results support our findings that a larger interparticle distance and higher aggregated fraction occur as transfer surface pressure is increased in the case of procedure A.

#### Procedure B. Controlled assembly of AgNCs by spontaneous adsorption on the surface

**Figure 2:**
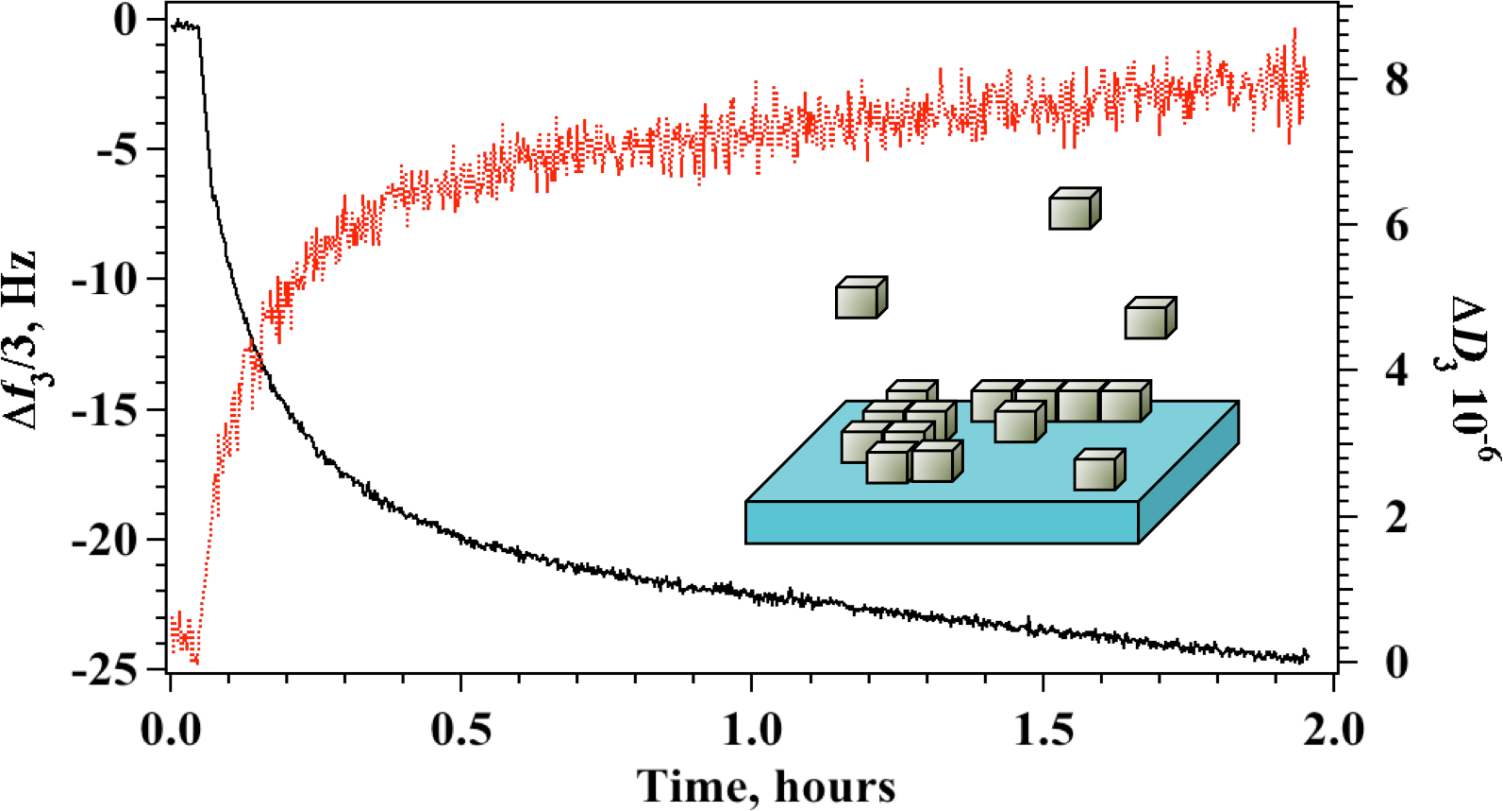
Procedure B. Formation of AgNC arrays by means of sequential physisorption. Δ*f*_3_/3 (black curve, left axis) and Δ*D*_3_ (red curve, right axis) as a function of time after a single addition of AgNC in the QCM measuring chamber.

Spontaneous self-assembly of AgNC on silicon oxide surfaces was monitored in situ by means of a quartz crystal microbalance as the nanocubes approach the substrate and adsorb on the surface. In this experiment, the change in frequency, Δ*F*, related to the adsorbed mass, and the change of the dissipation factor, Δ*D*, related to the viscoelastic properties of the adsorbed film, were measured simultaneously as a function of time. Aliquots of a 0.3 mg mL^−1^ dispersion of AgNC were added sequentially in the measuring chamber, additions were made after adsorption equilibrium was established and after the excess of AgNC in solution was removed by water rinsing. This procedure was repeated until no further changes were recorded, indicating that surface saturation was reached. Typical results for the change in normalized frequency and dissipation factor for the third harmonic obtained for a single addition are reported in Figure 2. The plot shows how both Δ*f*_3_/3 and Δ*D*_3_ change with time reaching a constant value only after 2 hours.

Interestingly, the kinetics of each adsorption step cannot be described by a simple Langmuir adsorption model but includes both a surface adsorption phase, with a significant change in Δ*f*_3_/3 and Δ*D*_3_ and a rearrangement step at the surface where the adsorbed mass changes only slightly. Along the rearrangement step, the dissipation factor does not change significantly and oscillates around 7 × 10^−6^, a value that is usually associated with quasi-rigid layers. As adsorption proceeds in the following additions, the dissipation increases to values characteristic of elastic films. The structural parameters obtained at surface saturation for the AgNC layer fabricated with procedure B are reported in Table 1 together with the thickness of the adsorbed layer that was extracted by the analysis of all overtones as reported elsewhere [40,46].

The spontaneous adsorption method yields nanostructures with average interparticle spacing of 119 nm and an average layer thickness of 32 nm, comparison with the corresponding parameters obtained for procedure A (see Table 1) shows that both the adsorbed mass and the average thickness obtained with this procedure are slightly smaller suggesting lower surface coverage. In both cases formation of complete bilayers can be excluded although the presence of domains of 3D clusters cannot be discarded at this stage. The value of the nanocube surface density is in agreement with other studies on AgNC. Sisco and Murphy [47] studied AuNCs electrostatically immobilized on 4-MBA SAM and obtained a NC surface density ranging from 5.5 to 22 cubes/μm^2^ with larger fraction of aggregated structures at higher surface densities (≈51%). Wang et al. [48] studied the formation of AgNC arrays by the dropping method as a function of AgNC concentration obtaining surface densities in the range 4.5–32.5 cubes/μm^2^, whereas LB transfer of the same nanocubes provided larger surface densities of 19 to 49 AgNCs/μm^2^ with increasing transfer surface pressure. The same authors also demonstrated that maximum Raman intensity of the R6G probe and enhancement factor are obtained for the large surface densities, i.e., 32.5 AgNC/μm^2^.

Absorbance spectra for the AgNC arrays obtained with procedure B were tentatively acquired for glass and silicon oxide substrates, respectively. Typical results on glass (see Supporting Information File 1, Figure S3) show that absorbance for samples obtained with procedure B is very small due the lower surface coverage and stability obtained through physisorption. Weak shoulders are visible at 412 nm and 500 nm, as in the case of LB transfer, but the long-range dipolar contribution at 600 nm is suppressed. Although the weak absorbance does not allow for an unequivocal conclusion, similar results were recently obtained also by Park et al. [49] for NC horizontal transfer on silicon oxide substrates from the liquid–liquid interface. In the absence of a suitable linking functionality the authors observed the formation of loosely-packed arrays that exhibited a modest red-shift of the position of the dipole surface plasmon mode and a very broad extinction profile from 420 to 1200 nm without significant features. Electron microscopy in the early work by Malynych and Chumanov [50] also revealed no long-range order within the assembly of nanoparticles when 2D array of 100 nm AgNP were assembled by direct adsorption of the NP onto modified surfaces.

### Adsorption of graphene oxide on AgNC arrays

**Figure 3:**
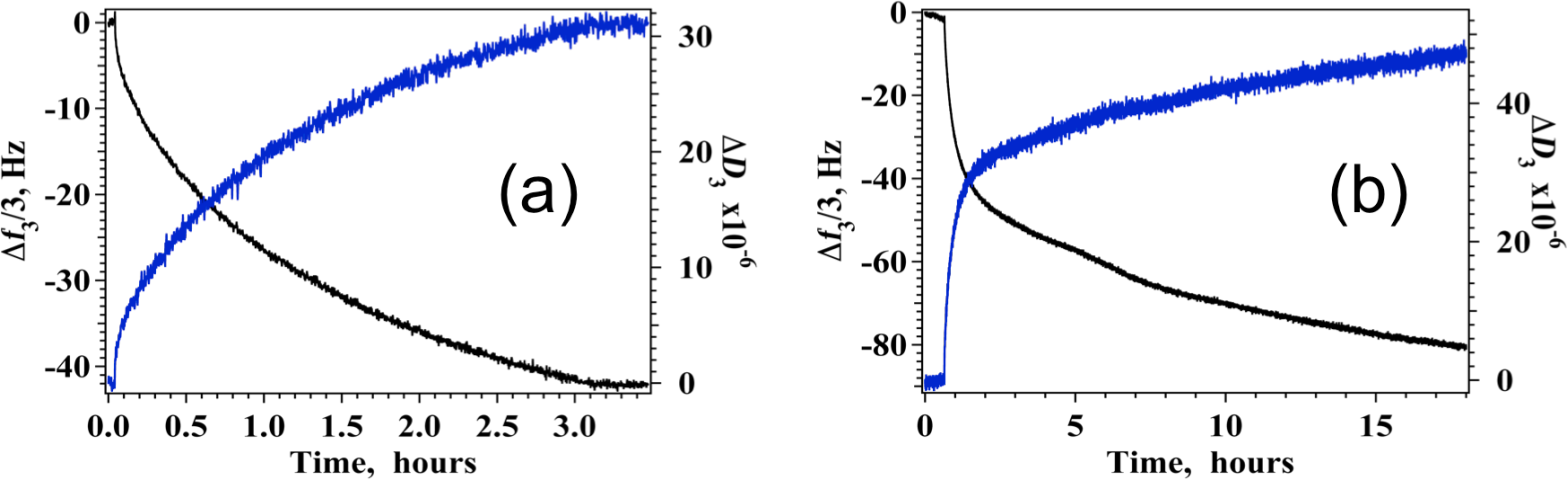
Change in Δ*f*_3_/3 (black curve, left *y*-axis) and Δ*D*_3_ (blues curve, right *y*-axis) upon addition of graphene oxide from aqueous solution to 1 LB layer of silver nanocubes transferred at 15 mN/m (a) and to silver nanocubes arrays assembled by spontaneous adsorption (b). In all experiments [GO] = 40 mg L^−1^ and *T* = 20 °C.

Different fabrication methods have been developed for SERS-active surfaces involving graphene derivatives and nanoparticles including metal evaporation, electrochemical deposition and layer-by-layer self-assembly techniques [51]. Zarbin's group [20] directly synthesized and assembled silver nanoparticle/graphene oxide nanocomposites at a water/toluene liquid–liquid interface whereas Wang et al. [52] proposed to assemble silver nanoparticles to graphene oxide sheets employing electrostatic interactions and a polymer, as adhesive agent, to impart greater stability against aggregation of AgNPs. Previous investigations are generally focused on spherical nanoparticles and only recently Fan et al. reported a work on single-particle SERS efficiencies of Ag nanooctahedra/GO hybrids built with drop-cast/adsorption method [19]. Most of these studies report on the fabrication of AgNC onto GO layers, without any control in situ of the AgNP packing density and of the morphology of the GO coverage. We adopted a different approach overlaying GO sheets on prepacked AgNC layers in the search for a reliable method that allows conjugation of AgNPs with desired morphologies (densities, sizes and shapes) with graphene oxide continuous covering. GO forms stable colloidal dispersions in water thanks to the presence of negatively charged carboxylic groups on its edges [53]. It has also been shown that GO flakes posses non-negligible surface activity that allows for the formation of spreading monolayers at the water–air interface and for its use in interfacial and flotation applications [54]. These features suggest that efficient coating of AgNC layers with graphene oxide may be obtained by direct adsorption from solution to the AgNC monolayer interface but a reproducible fabrication protocol of GO layers with controlled surface density and thickness necessitates a detailed knowledge of the mechanism and the kinetics of the process. To this end, the adsorption of graphene oxide flakes on the surface of the AgNC arrays was studied monitoring the change in adsorbed mass and thickness of the process by means of a quartz crystal microbalance. Preliminary studies demonstrated [21] that a decrease in frequency upon addition of GO was observed for GO bulk concentration as low as 4 mg L^−1^ with no mass loss upon water rinsing, the result indicates stable mass adsorption on the AgNC coated sensor surface even at low surface density. The same study [21] also evidenced that rapid increase in adsorbed mass was recorded up to 40 mg L^−1^, after this concentration further addition of GO produced only a smaller increase in GO surface density. Typical results obtained for sequential adsorption of a 40 mg L^−1^ solution of GO on AgNC assemblies obtained with procedure A are reported in Figure 3.

The plots in Figure 3a show a continuous variation in time of the adsorbed mass in a Langmuir-type behaviour [21]. Interestingly, the increase in mass was paralleled by an increase in Δ*D*_3_ value, which correlates with an increase in the viscoelastic properties of the adsorbed GO layer.

Analogously, QCM experiments were run to study the process of deposition of GO on top of AgNC layers assembled with procedure B, typical plots obtained for [GO] = 40 mg L^−1^ are reported in Figure 3b. Also in this case we observe a prompt decrease of frequency accompanied by an important increase in the dissipation factor, the behaviour of these two parameters with time is not continuous but reveals steps with different slopes that could be associated to a reorganization of the adsorbed GO flakes after arrival on the AgNC surface. For comparison we also recorded the adsorption behaviour of GO on bare silicon oxide surfaces, the results (Supporting Information File 1, Figure S4) revealed that GO adsorption is totally suppressed in the absence of the AgNC layer. Analysis of the experimental QCM results obtained for all overtones allowed for the determination of the surface density and thickness of the adsorbed GO layer, the values for the samples obtained with both procedures A and B at the same GO concentration, [GO] = 40 mg L^−1^, are summarized in Table 2.

**Table 2:** Main physico-chemical parameters for GO layer on AgNC arrays.

AgNC array packing procedure	Surface density, ng/cm^2^	δ, nm	Δ*D* × 10^−6^

procedure A (π_transfer_ = 15 mN/m)	1006	9 ± 1^a^ 8^b^	80^a^
procedure B	1598	8 ± 1^a^ 7^b^	125^a^

^a^Values extracted from analysis of QCM with dissipation monitoring measurements. ^b^Values extracted from analysis of AFM data.

The data reveal that similar GO surface densities are obtained for both procedures, although larger values are found in the case of AgNC arrays obtained for spontaneous adsorption. The average QCM thickness is similar in both cases and corresponds to values much larger than 1 nm, a value previously ascribed to single flat GO layer [55]. These results could be explained either in terms of multilayer stacking or, more likely, with the folding of the GO sheet on the surface, this latter process is in agreement also with the large dissipation factor observed. Dissipation changes are large in both cases indicating that, as the surface density of adsorbed GO increases, the GO sheets do not adsorb flat onto the AgNCs surface but behave as an elastic, flexible and continuous layer.

**Figure 4:**
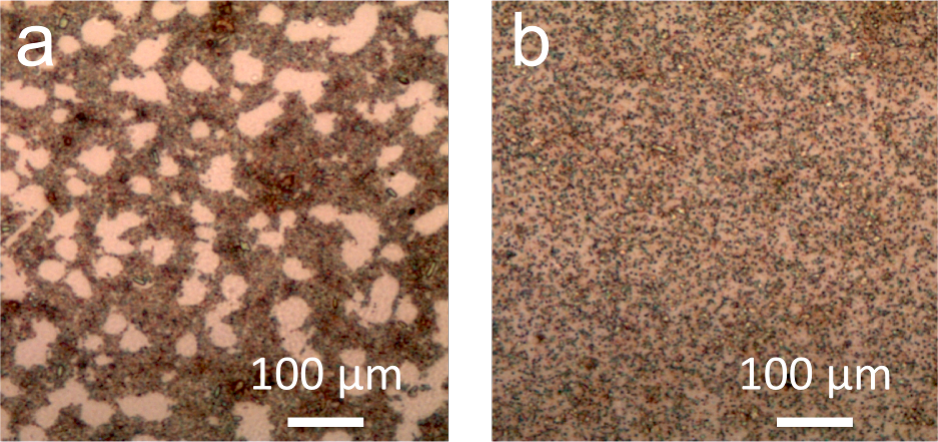
Optical images of GO-covered AgNCs prepared with procedure A (a) and B (b).

The viscoelastic behaviour of graphene derivatives appear particularly important also for an emergent class of new graphene-derived metamaterials as reported also in a recent paper [56] where McEuen and coworkers describe graphene kirigami, robust microscale structures with tunable mechanical properties. Possibility to obtain these structures relies on the ratio between the in-plane stiffness and out-of-plane bending stiffness: large values of this parameter translate in membrane-like material that more easily bend and crumple. Optical microscopy in reflection mode images (Figure 4) of the two systems disclose quite different morphologies although with similar overall nanocube surface coverage. GO/AgNC assembly obtained with the LB technique is characterized by densely packed regions alternated to empty surface areas whereas the entire sensor surface is more homogeneously covered for GO/AgNC obtained with procedure B.

**Figure 5:**
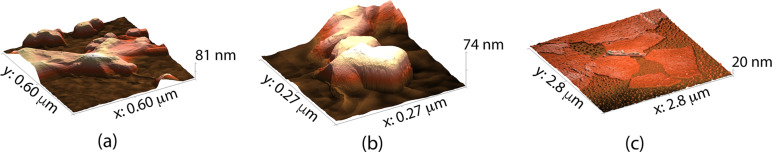
(a) AFM image of GO-covered silver nanocubes obtained from Langmuir–Blodgett transfer at 15 mN/m. (b) AFM image of GO-covered silver nanocubes obtained from direct sequential adsorption. (c) AFM image of the GO flakes on silicon oxide surfaces.

The larger amount of GO obtained with procedure B (see Table 2) is therefore strictly related to the smaller fraction of bare silicon oxide surface exposed. In both cases GO adsorbs through interactions with the silver surface anchoring exclusively to the outer face of the nanocube, with the large GO flakes extending and folding on the remaining nanocubes of the clusters or on the SiO_2_ surface. This rationale is supported also by high resolution AFM images reported in Figure 5, the samples exhibit very similar structuring of the GO sheet over the AgNC assembly.

Figure 5c refers to graphene oxide deposits on silicon oxide obtained by simple drop casting followed by evaporation and shows, as expected, the presence of irregularly shaped sheets of lateral dimension ranging from a few nanometres to micrometres with nonuniform thickness ranging from 1.0 nm for single layers to 3.6 nm for GO terraces where the flakes partly overlap. AFM data confirm that folding of GO is prompted only by specific GO–AgNC interactions that anchor the larger GO sheets extending the coverage also on the uncovered surface. AFM analysis (see Table 2) revealed that adsorbed GO has an average thickness of 7 nm for both procedures. Such high thickness values are in accordance with the formation of folded geometries of single GO sheets after anchoring to the nanoparticle surface. Nevertheless, AFM measurements reveal a smaller thickness compared to QCM for the same samples. This was expected since QCM values are always affected by the presence of water when hydrophilic substance are involved, as is the case of GO. Moreover, the presence of multiple GO folds offers inner surfaces where bound water can be confined, a phenomenon that escapes AFM detection but may be promptly revealed by gravimetric measurements.

Visible spectra for GO covered AgNCs on silicon oxide were collected in reflection mode using an integrating sphere for samples obtained with both procedure A and B are reported in Figure S5 of Supporting Information File 1. The spectra show that the dipole long-range coupling is broader and red-shifted for both packing procedures, although a red shift in the presence of GO was expected due to the higher refractive index of GO, the visible spectra of GO covered LB films evidenced only a modest red-shift to 670 nm. The larger shift observed in the present study may be explained considering that the use of the integrating sphere allows for the collection of reflected light coming from all angles and deriving from excitation at different incident angles with respect to the interface, therefore suggesting that in-plane and out-of-plane ordering of the nanocubes obtained with the two procedures are significantly different. Although the nanocubes are in a single layer with controlled interparticle distance they have a random rotational alignment perpendicular to the substrate and give an average response that includes all alignments. A strong angle dependence of the extinction and reflection spectra was observed also by other authors [57] and precludes a clear-cut spectral assignment at this stage.

#### SERS experiments

We analysed the SERS performances of the GO/AgNCs assemblies fabricated using procedure A (LB film of AgNCs) and B (sequential adsorption of AgNCs) and the results were compared with those obtained on pristine AgNCs LB films, excitation wavelength was fixed at 638 nm for both systems for the sake of comparison. The SERS experiments were conducted by using adenine as model probe because of its well-known SERS response and potential to establish interactions with both noble metal and graphene surfaces [58].

**Figure 6:**
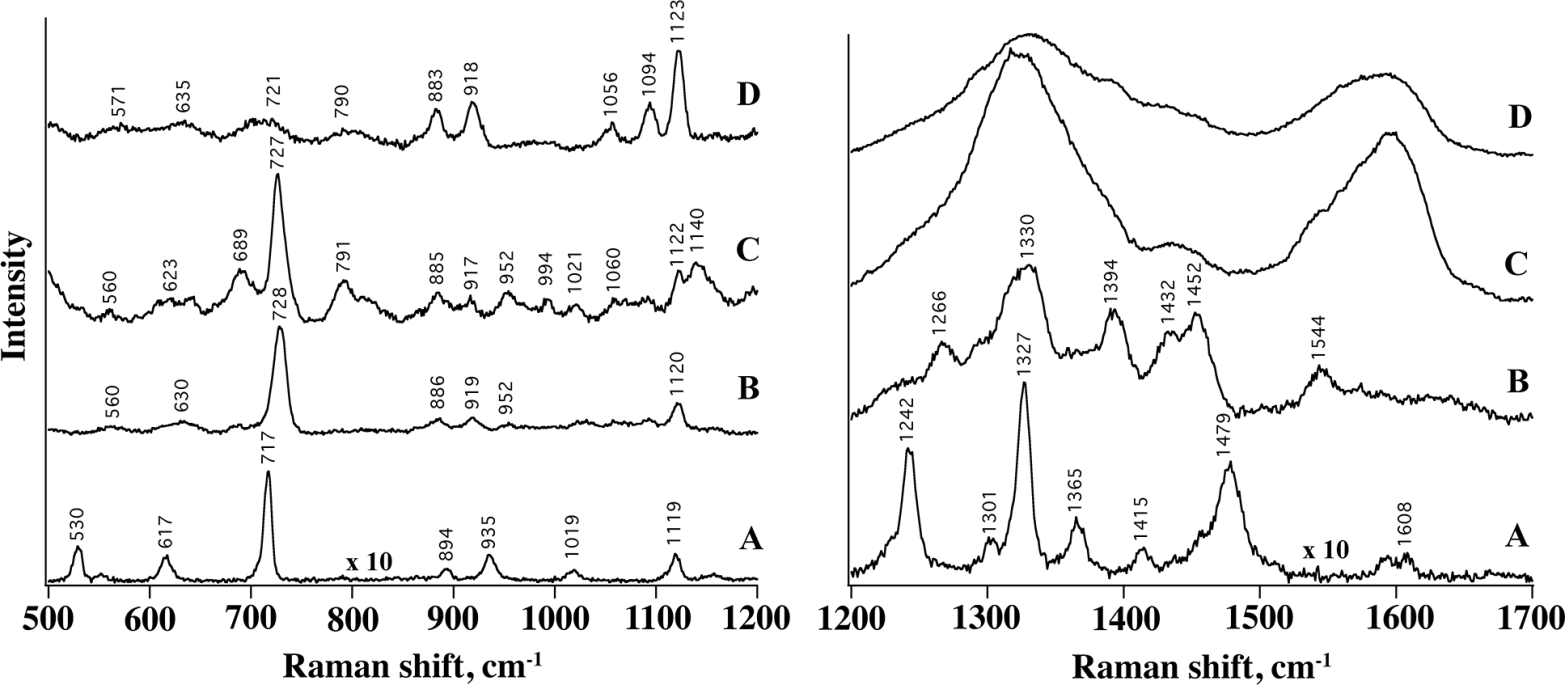
SERS spectra of Adenine (9 × 10^−7^ M) adsorbed on single LB layer of AgNCs (B), on GO/AgNCs obtained with procedure A (C) and with procedure B (D). Raman profile from adenine powder is also shown (A). Left: 500–1200 cm^−1^ Raman shift. Right: 1200–1799 cm^−1^ Raman shift.

In Figure 6 the SERS spectra of adenine adsorbed from a 9 × 10^−7^ M incubation solution on AgNCs (B) and hybrid GO/AgNCs substrates (C, D) is displayed. Overall, the signals exhibit similar spectroscopic features (frequencies, S/N ratio, intensity), which suggests the occurrence of comparable interactions between the probe and the different substrates. Interestingly, the SERS spectrum of adenine features more detailed information in the presence of a GO layer, allowing even weaker Raman peaks (e.g., within the 750–1200 cm^−1^ region) to be resolved (Figure 6A, system C) possibly due to more favourable interactions between the adenine molecules and the substrate and/or a higher local concentration of adenine molecules retained by the GO layer as compared to those adsorbed on the naked nanoparticle substrates.

The SERS spectra closely resemble the Raman profile of adenine (A) in the region between 500 and 1200 cm^−1^, including the intense ring breathing peak at 730 cm^−1^, which undergoes a 10 cm^−1^ blue shift ascribed to the interaction with the metal and GO surfaces. This peak is markedly weaker in the case of GO/AgNCs obtained with procedure B, which we attributed to a looser distribution of AgNPs and in turn to a reduced interparticle electromagnetic coupling and number of hot spots within the illuminated sample volume. Here the most intense bands observed are assigned to residual PVP molecules on the AgNCs surface (Supporting Information File 1, Figure S6).

The characteristic GO bands centred at 1601 cm^−1^ (G band) and at 1365 cm^−1^ (D band), and corresponding to the tangential stretching mode of the E_2g_ phonon of sp^2^ atoms and to the breathing mode of κ-point phonons [59], respectively, dominate the Raman shift region between 1200 and 1700 cm^−1^ of the hybrid substrates (Figure 6 right) at the expense of the adenine signals. These bands are electromagnetically 50-fold enhanced by the underlying silver layer and this effect appears more pronounced for the GO/AgNCs assemblies obtained with procedure A (Supporting Information File 1, Figure S7).

**Figure 7:**
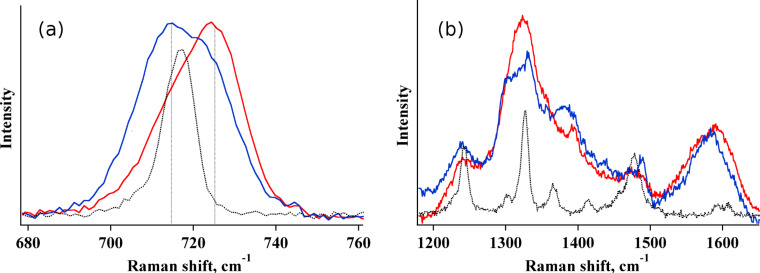
SERS spectra of Adenine (9 × 10^−4^ M) adsorbed on GO/AgNCs obtained with procedure A (red curve) and with procedure B (blue curve). The Raman spectrum (×10) of adenine powder is also reported (black curve).

When the samples are incubated with a more concentrated adenine solution, i.e., [adenine] = 9 × 10^−4^ M, a contribution from pure Raman occurs (see Figure 7) and overlaps with the SERS signals and this effect is more pronounced for GO/AgNCs samples obtained with procedure B. For example, the SERS contribution to the 730 cm^−1^ band reduces from 65% to 31% passing from type A to type B GO/AgNCs nanoarrays (Figure 7a), which again highlights how the non-homogenous assembly obtained with the Langmuir–Blodgett technique confers superior SERS activity compared with the samples obtained by spontaneous adsorption. Accordingly, the Raman signals of adenine can be detected against the prevailing GO bands in the GO/AgNCs substrate obtained by procedure B, which is not the case of that obtained by procedure A (Figure 7b).

**Figure 8:**
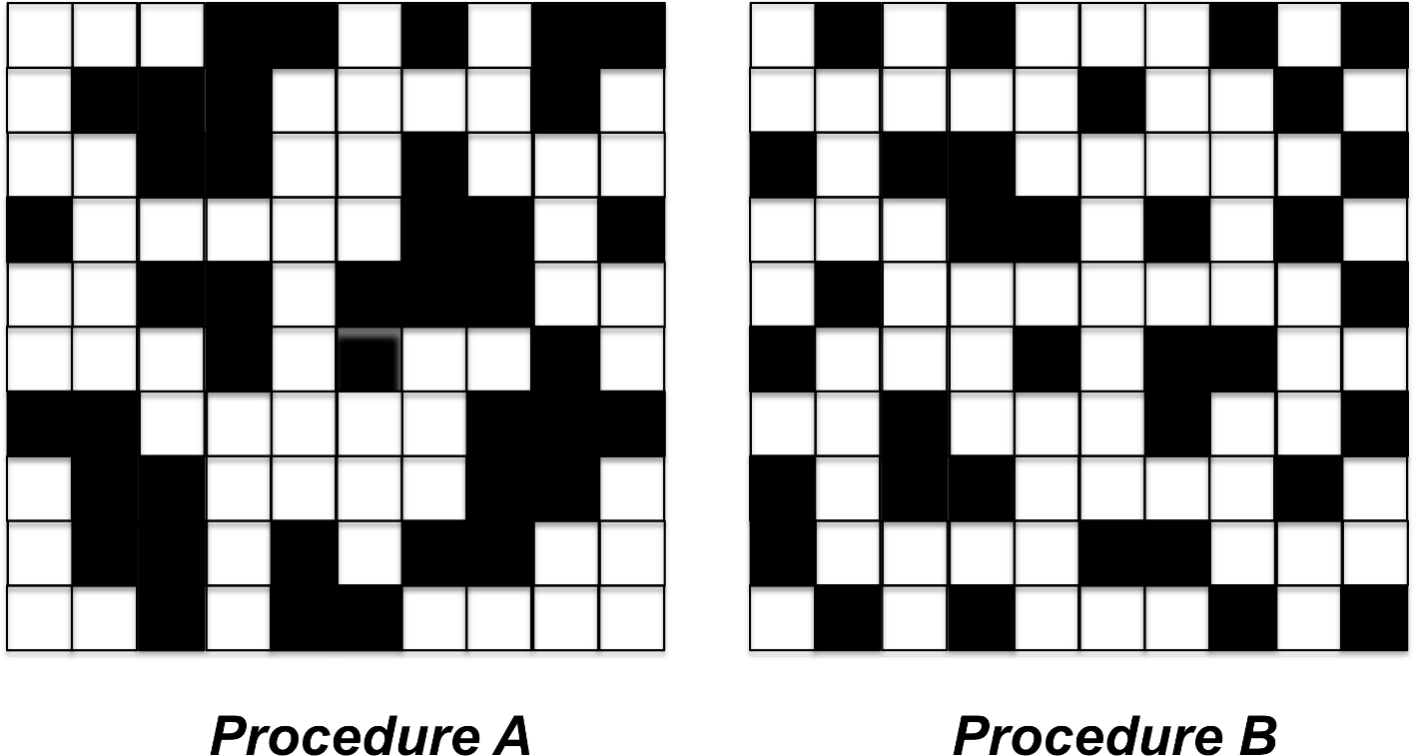
Checkboard model for AgNCs arrays on SiO_2_ obtained with procedure A (surface density = 41 NC/μm^2^) and procedure B (surface density = 35 NC/μm^2^).

The spectra in Figure 7 suggest that procedure A is the preferred approach for the production of reproducible SERS-active substrates although larger GO coverage and uniformity are obtained when AgNCs are assembled by spontaneous adsorption. This behaviour can be rationalized on the basis of the different clusters distribution obtained with the two procedures, as shown in the cartoon in Figure 8.

The scheme in Figure 8 describes a check board representation of the two systems, the overall number of nanocubes correlates with the experimental surface coverage reported in Table 1. Although the average intercube distance found from QCM data is similar in the two systems, AFM data reveal that a large fraction of the nanocubes are grouped together in the case of procedure A with an average spacing of a few nanometers. Conversely, self-assembled nanocubes obtained from procedure B are uniformly dispersed as single nanocubes with larger spacing and only sparse NCs clusters. In the example reported in Figure 8, we can estimate that in the case of procedure B only 14% of nanocubes are closely packed but this fraction increases to 38% for samples obtained with procedure A. This picture may explain SERS results considering that the presence of a large fraction of AgNCs clusters with small interparticle distance permits the creation of an efficient hot spot distribution. Additionally, recent experiments on localized surface plasmon emission via delayed femtosecond laser pulses confirmed that small clusters lead to a plasmonic response that provides the highest peak intensity [60].

## Conclusion

Here we stress how a different packing geometry between assembled silver nanocubes can affect the SERS signal detected on the surface of a GO covering layer used to improve the SERS response. A detailed QCM study revealed that GO coating of the nanocubes resulted in similar GO surface coverage and thickness for different AgNC arrays as long as their surface densities are equivalent. Structural characterization of the samples evidenced that GO/AgNCs arrays exhibit a strikingly different distribution of the GO veiled nanocubes that is reflected in distinct SERS response. While a spontaneous physisorption of cubes favours the formation of homogeneously distributed nanoparticle arrays, the method severely limits the construction of reproducible SERS-active substrates due to a large fraction of cubes with a large separation distance. Instead, a Langmuir–Blodgett transfer of a floating monolayer of silver cubes produces a large fraction of particle clusters with small interparticle distance, which generates an efficient hot spot distribution.

## Experimental

**Materials.** Ethylene Glycol (EG, ≥99%) was obtained from Scharlab. Sodium sulfide nonahydrate, PVP (*M*_w_ = 55000), silver nitrate and GO solution (4 mg mL^−1^) were obtained by Sigma-Aldrich. Aqueous solutions were prepared using ultrapure Milli-Q water. Silicon wafers (n-type, no dopant) were purchased from Sigma-Aldrich.

**Synthesis of AgNCs.** EG (10 mL) was placed into a flask and heated under magnetic stirring in an oil bath at 150 °C for 1 h under a nitrogen flow. Then, 0.175 mL of a 0.72 mg mL^−1^ sodium sulfide solution and 3.75 mL of a 20 mg mL^−1^ PVP solution in EG were subsequently added to the flask. The flask was thermostated for additional 10 min, until a temperature of 150 °C was again established. A silver nitrate solution (1.25 mL) in EG with a concentration of 48 mg mL^−1^ was added dropwise to the reaction flask at a rate of approximately 1 mL min^−1^. The reaction was stopped after 40 min by placing the flask in an ice-bath and by adding 30 mL of acetone. The nanoparticles were then centrifuged at 10000*g* for 30 min and then dispersed in ethanol or chloroform by using an ultrasonic bath. The washing procedure was repeated at least three times in order to ensure the complete removal of the reagents. The suspensions of AgNCs thus obtained were stored in centrifuge tubes at −20 °C.

**Transmission electron microscopy measurements.** TEM micrographs of the particles were acquired with a Philips CM-12 microscope running at 100 kV.

**Langmuir–Blodgett film preparation.** Langmuir monolayers were prepared in a symmetric compression trough (KSV3000 trough, KSV Instruments Ltd., Finland) filled with Milli-Q water (resistivity = 18 MΩ cm, pH 5.6 at 20 °C). A suspension of AgNCs in chloroform (volume = 1.65 mL, [AgNCs] = 3.1 mg mL^−1^) was deposited dropwise over the water surface and 40 min were allowed for solvent evaporation before starting the compression. Surface pressure was measured with a platinum Wilhelmy plate as a function of the surface area at *T* = 20 ± 0.5 °C (Haake thermostatic bath, Germany). Continuous spreading isotherms and hysteresis cycles were obtained using the same barrier speed of 20 mm min^−1^ in both directions. The reported results are the average of at least three independent measurements. Langmuir–Blodgett films were transferred, after area cycling, onto quartz slides and SiO_2_-covered QCM quartz sensors by vertical dipping at a rate of 2 mm min^−1^ at several target surface pressures in the range 5 mN/m ≤ π ≤ 20 mN/m. All substrates were rinsed with ethanol and treated in a plasma cleaner immediately before deposition; different substrates were simultaneously coated by the same AgNC layer.

**Quartz crystal microbalance measurements.** QCM experiments with impedance monitoring were performed on a QCM-Z500 (KSV Instruments Ltd) equipped with a thermoelectric (TE) module (Oven Instruments). The resonant frequency shift and the change in energy dissipation of a SiO_2_-coated AT-cut 5 MHz quartz microcrystal were simultaneously measured at its resonant frequency and at the third, fifth, seventh, ninth and eleventh overtones. The temperature of the measuring cell was kept constant at 20 °C with a Peltier element connected to the TE module. For thin, uniform and rigid or quasi-rigid films in solution, the resonant frequency is linearly proportional to the mass density of the deposited film according to the Sauerbrey equation; for thicker or less rigid films a more complex analysis must be undertaken since the resonance frequency is affected not only by the mass attached to the surface but also by the viscoelastic properties of the adsorbed layer. Frequency and admittance data were simultaneously recorded and taken into consideration for the analysis. The QCM experimental data were analysed by means of the commercial QCMBrowse analysis software to estimate adsorbed mass and film thickness [45].

**AFM measurements.** Non-contact AC mode atomic force microscopy (AFM) images were acquired in air using a PicoSPM microscope equipped with an AC-mode controller (Keysight Technologies, Inc formerly Molecular Imaging). For optimal resolution rectangular non-contact gold coated cantilever were used (model Hi'Res-C14 from MicroMash – http://www.spmtips.com), with typical resonance frequency of 160 kHz, and 1 nm tip radius. The nanocube dimensions were measured from the height statistics in the topographic AFM images. Image processing and pseudo 3D rendering was performed using Gwyddion 2.30 SPM data visualization tool (http://gwyddion.net/).

**UV–vis measurements.** UV–vis spectra of the nanocube suspension in ethanol and of monolayers deposited on quartz substrates were recorded using a Jasco V-6 UV–vis–NIR spectrophotometer with 1 nm slit and 200 nm min^−1^ scan rate. Reflection spectra were the average of 10 scans.

**SERS measurements.** Raman measurements were performed at room temperature on an XPlora Horiba MicroRaman with a 638 nm laser as excitation source. We used a 100× objective with accumulation times of 10 s per spectrum and a 70 µW power on the sample. The SERS substrates were pre-immersed in a 9 × 10^−4^ M or 9 × 10^−7^ M adenine solution for two hours to ensure that adsorption equilibrium was reached. The samples were rinsed with deionized water and dried under nitrogen flux before each SERS measurement.

## Supporting Information

The Supporting Information features compression–expansion cycles and transfer ratios for LB transfer; absorbance and reflectance spectra for AgNC and GO/AgNC arrays for procedure A and B; QCM data for graphene oxide adsorption on bare silicon oxide surfaces; and SERS and Raman spectra for adenine, PVP and GO.

File 1 Additional thermodynamic and spectroscopic characterization.
