# Supplementary material for: Controlled graphene oxide assembly on silver nanocube monolayers for SERS detection: dependence on nanocube packing procedure
Source: Beilstein J Nanotechnol. 2016 Jan 6;7:9–21. doi: 10.3762/bjnano.7.2 (PMC4734306; doi:10.3762/bjnano.7.2)
Supplement: File 1 — Additional thermodynamic and spectroscopic characterization. [file Beilstein_J_Nanotechnol-07-09-s001.pdf]

Supporting Information

for

Controlled graphene oxide assembly on silver nanocubes monolayers for SERS detection: dependence on nanocubes packing procedure

Martina Banchelli[1], Bruno Tiribilli[2], Roberto Pini[1], Luigi Dei[3], Paolo Matteini[*1] and Gabriella Caminati[*3]

Address: [1]Institute of Applied Physics, National Research Council - Via Madonna del Piano 10, I-50019 Sesto Fiorentino, Italy, [2]Institute for Complex Systems, National Research Council, Via Madonna del Piano 10, I-50019 Sesto Fiorentino, Italy and [3]Department of Chemistry and CSGI, University of Florence, Via della Lastruccia 3–13, I-50019 Sesto Fiorentino, Italy

E-mail: G. Caminati - gabriella.caminati@unifi.it; P.Matteini - p.matteini@ifac.cnr.it

* Corresponding author

Additional thermodynamic and spectroscopic characterization

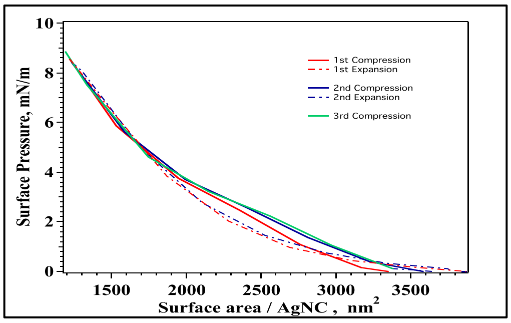

Figure S1: Hysteresis cycle for PVP capped silver nanocubes monolayers at water–air interface.

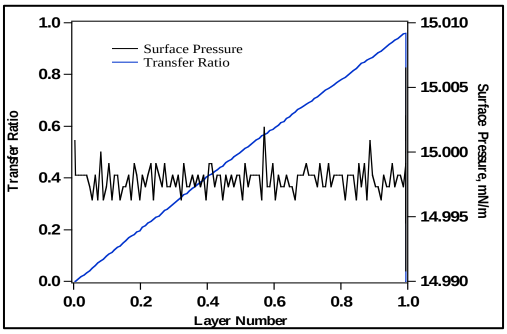

Figure S2: Transfer ratio (left axis) and transfer surface pressure (right axis) for Langmuir–Blodgett transfer of one AgNCs layer on glass substrate.

Similar results were obtained also for the other examined substrates, i.e., quartz and silicon oxide.

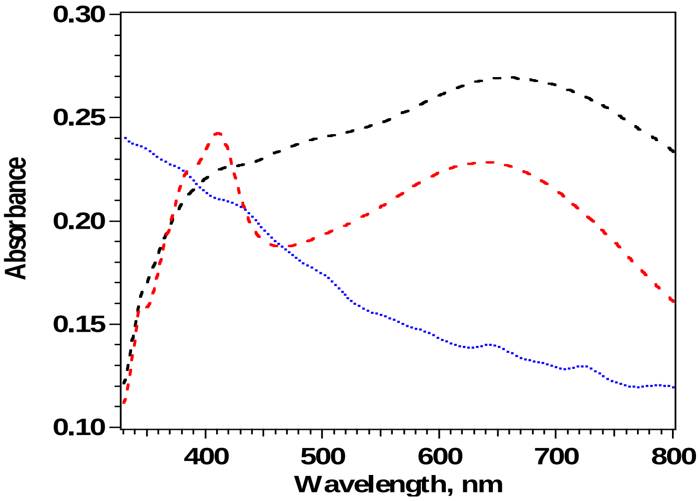

Figure S3: Absorbance (blue, dotted line) of AgNC arrays prepared with procedure B. Absorbance of AgNC arrays prepared with Procedure A with $\pi_{transfer}$ = 20 mN/m (black dashed line) and $\pi_{transfer}$ = 15 mN/m (red dashed line).

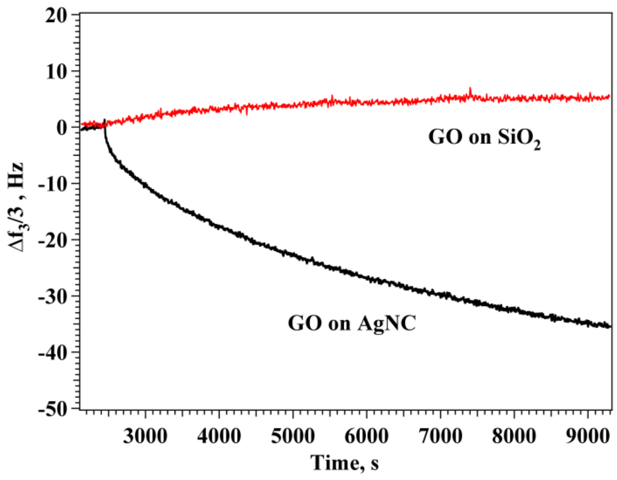

Figure S4: Change in $\Delta f_3/3$ and ΔD_3 upon addition of graphene oxide from aqueous solution to 1 LB layer of silver nanocubes transferred at 15 mN/m onto silicon oxide coated QCM quartz sensor and on bare silicon oxide QCM sensor.

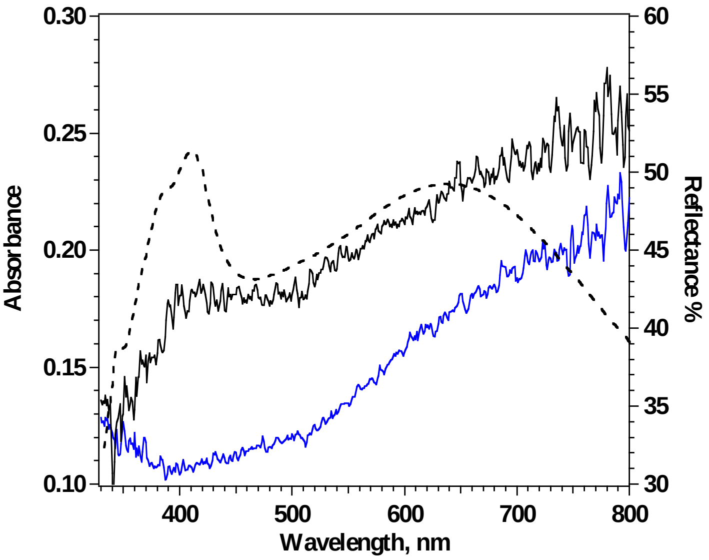

Figure S5: Reflection spectra of GO/AgNC arrays prepared with procedure A (black solid line) and procedure B (blue, solid line). Absorbance of AgNC arrays prepared with procedure A with $\pi_{transfer}$ = 15 mN/m (black dashed line).

As previously reported, the nanocubes exhibit a strong angle-dependent reflection of light from a dielectric surface as the angle of incidence is varied. The dependence comes from the plasmonic nature of the nanocubes. Minima in the reflection spectra at 400 nm for 40 nm cubes corresponding to their plasmonic resonances were also found [1].

[1] Bottomley A. and Ianoul A. *J. Phys. Chem. C* **2014**, *118*, 27509−27515.

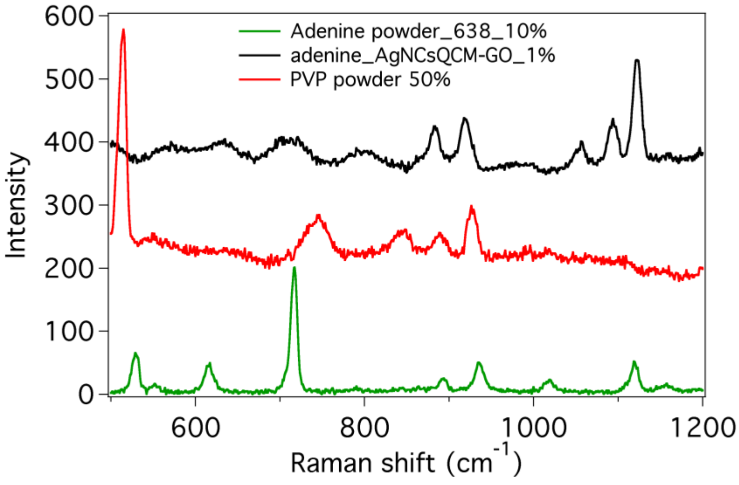

Figure S6: SERS spectra for PVP powder (red curve), adenine powder (green curve).

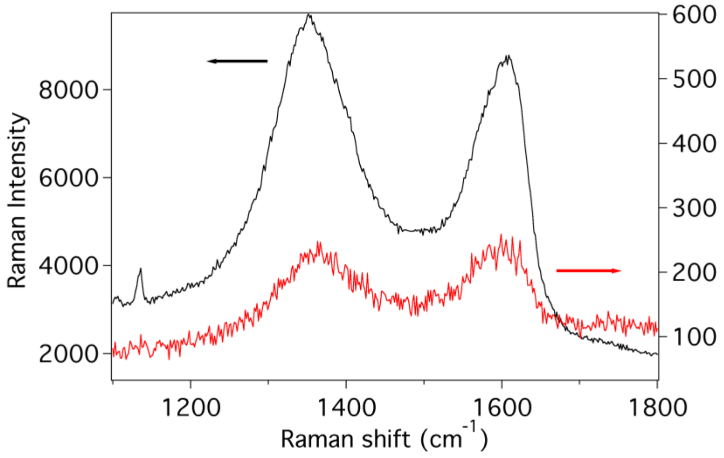

Figure S7: Raman spectra for GO (red curve) and GO adsorbed on AgNC arrays (black curve).
